# The Role of Ghrelin in Anorexia Nervosa

**DOI:** 10.3390/ijms19072117

**Published:** 2018-07-20

**Authors:** Martha A. Schalla, Andreas Stengel

**Affiliations:** 1 Charité Center for Internal Medicine and Dermatology, Department for Psychosomatic Medicine, Charité-Universitätsmedizin Berlin, Corporate Member of Freie Universität Berlin, Humboldt-Universität zu Berlin, and Berlin Institute of Health, 12203 Berlin, Germany; martha.schalla@charite.de; 2 Department of Psychosomatic Medicine and Psychotherapy, Medical University Hospital Tübingen, 72076 Tübingen, Germany

**Keywords:** animal model, body weight, brain-gut axis, drug, eating disorder, food intake, gut-brain axis, hormone, hunger, metabolism, psychosomatic, satiety

## Abstract

Ghrelin, a 28-amino acid peptide hormone expressed in X/A-like endocrine cells of the stomach, is the only known peripherally produced and centrally acting peptide that stimulates food intake and therefore attracted a lot of attention with one major focus on the treatment of conditions where an increased energy intake or body weight gain is desired. Anorexia nervosa is an eating disorder characterized by a pronounced reduction of body weight, a disturbed body image and hormonal alterations. Ghrelin signaling has been thoroughly investigated under conditions of anorexia nervosa. The present review will highlight these alterations of ghrelin in anorexia and discuss possible treatment strategies targeting ghrelin signaling. Lastly, gaps in knowledge will be mentioned to foster future research.

## 1. Introduction

Ghrelin was discovered in 1999 by Kojima and colleagues as an endogenous ligand of the growth hormone secretagogues receptor 1a (GHSR1a) stimulating the release of growth hormone (GH) from the pituitary [1] leading to the release of insulin-like growth factor 1 (IGF-1) [2]. Interestingly, GHSR1a is expressed, among others, in the arcuate nucleus, colocalized in neurons expressing Agouti-related peptide (AgRP) and neuropeptide Y (NPY) that regulate food intake [3]. Ghrelin was found to be secreted from oxyntic glands in the gastric fundus [1] and the subsequent description of ghrelin’s effects on food intake, glucose metabolism and body weight followed soon thereafter [4].

Ghrelin is derived from preproghrelin and activated by acylation, namely by the attachment of a fatty acid side chain to its serine 3 residue catalyzed by the ghrelin-*O*-acyl transferase (GOAT) [5]. Interestingly, another peptide hormone is derived from this precursor, namely obestatin [6]. However, since the initially proposed effects were not reproducibly by several other groups as well as by the original authors, its role is controversially discussed [7] Therefore, we will not focus on obestatin in the present review.

Acylated ghrelin is able to bind to and activate the GHSR1a leading to, among other effects, a stimulation of food intake [8], reduction of insulin secretion resulting in hyperglycemia [9] and stimulation of gastric motility [10], whereas desacyl ghrelin—long thought to represent a non-active form of ghrelin—is assumed to counterbalance the orexigenic effect of acyl ghrelin [11,12]. It is to note that desacyl ghrelin does not bind to the GHSR1a at physiological but acts as agonist at supraphysiological levels [13,14]. Further supporting a counterbalancing effect, mice overexpressing desacyl ghrelin were shown to consume less food associated with a loss of body weight and fat mass as well as reduced linear growth [15].

Ghrelin is not only produced in X/A-like cells of the stomach [16], but also in pancreatic cells [17], the intestine as well as to lesser extent in kidney, liver, spleen, heart, lung, gonads, skin and adipose tissue [18]. Similarly, also GOAT is expressed in the stomach, pancreas, intestine, hypothalamus, ovary, placenta, muscle, heart and adrenal glands and has been detected in the circulation as well [19,20,21,22,23]. Since the lumen of the stomach provides the medium-chain fatty acids necessary for ghrelin’s acylation, the main source of acylated ghrelin is assumed to be in the stomach [24]. The X/A-like cells are stimulated through β_1_ adrenergic [25], GIP (gastric inhibitory polypeptide/glucose-dependent insulinotropic peptide) [26], alpha transducin and gustducin and Tas1R3 receptor pathways [27], and inhibited via somatostatin receptors [28,29].

Besides its stimulatory effect on food intake, in the last two decades ghrelin was shown to be implicated in various functions giving rise to a pleiotropic mode of action [30] with stimulatory effects on gastrointestinal motility, lipogenesis, blood glucose levels and inhibitory effects on blood pressure and luteinizing hormone as well as follicle stimulating hormone [31]. It is also to note that ghrelin exerts protective and/or therapeutic effects in rodents via the GHSR1a in different tissues including the spinal cord [32], heart [33], kidney [34], pancreas [35,36,37,38], ulcerated oral mucosa [39,40], stomach and duodenum [41,42,43] as well as colon [43,44,45].

Early on, it was described that ghrelin is regulated in a meal-dependent fashion with a fasting/pre-prandial increase of gastric *ghrelin* and *GOAT* mRNA expression [23] resulting in increased circulating ghrelin levels [46] and a decrease after the meal with lipids being especially potent to reduce circulating ghrelin [47,48]. Interestingly, low gastric pH increases the release of desacyl ghrelin from the stomach [49], probably to initiate the termination of food intake. Moreover, also chronic alterations of body weight were shown to affect circulating ghrelin levels with a decrease observed under conditions of overweight/obesity [50] and an increase in patients with cachexia [51] as well as anorexia nervosa [52] as detailed below.

Peripheral ghrelin’s orexigenic effect is thought to be mediated centrally, namely after crossing the blood-brain barrier via binding to its receptor located on NPY- and AgRP-expressing neurons in the arcuate nucleus initiating the mTORC1/S6K1 (mechanistic target of rapamycin complex 1/p70 ribosomal protein kinase 1) pathway [53,54]. Moreover, ghrelin can also bind to vagal afferents that signal to the brainstem to induce an orexigenic effect [55]. Consequently, ghrelin is a hallmark hormone of the gut-brain axis and early on attracted attention as possible target in the treatment of conditions where stimulation of food intake or weight gain is desired.

Patients suffering anorexia nervosa (AN) intentionally loss body weight by reducing food intake, hyperactivity, self-induced vomiting and abuse of laxatives [56]. The body mass index (BMI) defined as ≤17.5 kg/m^2^ and can reach life-threatening values under 12; consequently, the disease has a high mortality between 5% and 20% [57]. The rising prevalence of eating disorders worldwide [58] underlines the necessity to expand the knowledge about their pathophysiology. Recent studies give rise to an important role for ghrelin in the pathophysiology and clinical course of AN.

Therefore, the present review will describe the alterations of ghrelin under conditions of AN and ghrelin’s putative role as a drug candidate in the treatment of AN. Moreover, gaps in knowledge will be addressed to stimulate future research.

## 2. Alteration of Ghrelin in Anorexia Nervosa

### 2.1. Basal Circulating Total Ghrelin Levels

In 2001 a significant difference between fasting plasma ghrelin levels in anorexic patients compared to age-matched healthy controls was observed for the first time [52]. Ghrelin levels were elevated in AN patients and reduced after therapeutically induced increase of BMI resulting in a negative correlation between BMI and ghrelin [52]. Subsequent studies corroborated these findings [59,60], alterations also observed in an animal model for anorexia nervosa, activity-based anorexia (ABA) [61,62]. Since ghrelin correlates positively with the extent of physical activity [63], hyperactivity is likely to play a role in the elevation of ghrelin.

Additionally, a negative correlation between fasting ghrelin and insulin levels was observed [64]. Another study extended these findings by the observation of negative correlations between percent body fat and serum cholesterase in AN as well as a positive correlation with serum amylase [65]. Interestingly, this study, using a self-developed radioimmunoassay, differentiated between anorexic patients of the binge eating/purging type (AN-BP) and the restricted type (AN-R), showing significantly higher fasting mean plasma levels of ghrelin in those with binge eating/purging behavior, pointing towards an effect of bingeing and purging on ghrelin release/concentration [65], additionally illustrating a positive correlation between frequencies of binge/purge cycles with plasma ghrelin concentration [66]. In contrast, a negative correlation was observed between frequency and severity of binge eating and purging behavior as measured by BULIT-R (bulimia test) total scores and ghrelin concentrations, and lower plasma ghrelin concentrations in patients with AN-BP compared to those suffering from AN-R [67]. These opposing results may result from different BMI values of included subjects (13.7 ± 1.9 and 13.6 ± 1.5 in [65], 13.9 and 14.4 in [66] vs. 16.0 ± 2.4 in [67]). It has to be noted that the results were obtained using different radioimmunoassays, self-developed [65] or commercially purchased [67] possibly also contributing to the different levels observed. An additional study included emergently hospitalized AN patients, showing higher ghrelin levels in those patients compared to patients with AN-BP, AN-R and age-matched healthy female controls [68]. Further supporting a link between purging and ghrelin, examinations using the Three-Factor Eating Questionnaire demonstrated a positive correlation between ghrelin secretion during the cephalic phase and the tendency to lose control over eating in AN [69].

Following up on these findings in studies comparing AN to normal weight controls, in 2003 a study compared AN patients with BMI-matched healthy subjects showing that AN patients had doubled fasting and 24-h plasma ghrelin levels compared to constitutionally lean subjects [70], giving rise to a role of ghrelin in the development or maintenance of AN. Another study even showed reduced ghrelin levels in constitutionally lean subjects compared to normal weight controls, while ghrelin levels in AN were increased [71]. Moreover, AN subjects also displayed higher ghrelin levels compared to cancer patients developing cachexia [72]. The underlying mechanism responsible for this specific up-regulation remains to be unraveled.

In 2005, a study examined ghrelin’s secretion in adolescents in detail, demonstrating that its concentration is not only higher in AN but also its nadir as well as total area under the curve over 12 h of nocturnal sampling were elevated [73]; this period of time was chosen since ghrelin rises physiologically under conditions of fasting such as during sleep, likely to stimulate subsequent food intake [53]. In addition, secretory burst amplitude and burst mass were increased in AN resulting in higher pulsatile and total ghrelin secretion [73]. Hereby, fasting ghrelin was related toGH burst frequency with predictive value [73]. Nutritional markers such as BMI and body fat correlated with nadir and post-glucose but not fasting ghrelin levels, whereas those were negatively related to fasting insulin, insulin resistance, leptin, insulin-like growth factor (IGF-1) and HOMA-IR (homeostasis model assessment of insulin resistance) [73,74]. Low levels of IGF-1 and insulin are likely related to low energy and nutritional status in AN, since IGF-1 stimulates cell growth whereby an adequate energy state is necessary [75], and insulin is responsible for anabolic processes suppressed due to the lack of energy [76]. Furthermore, ghrelin was associated with total T_3_ (triiodothyronine) and luteinizing hormone levels, but only nadir ghrelin independently predicted cortisol burst frequency [73]. A different study observed this positive correlation between ghrelin and cortisol only in female AN patients but not in female healthy controls [67], likely reflecting/contributing to the stress component of the disease.

### 2.2. Expression of Ghrelin

In immunohistological expression analyses of pituitary from healthy humans and anorexic subjects, ghrelin was localized mainly in somatotrophs [77]. Controls showed only limited immunoreactivity, whereas in the tissue of anorexic subjects immunoreactive cells were observed in nerve fibers and Herring bodies of the posterior pituitary and pituitary stalk [77]. Moreover, in contrast to controls ghrelin was additionally apparent in the anterior pituitary [77]. Higher central ghrelin signaling is likely a compensatory mechanism to stimulate food intake and body weight gain in AN. It is important to note that human data are lacking so far that describe the alterations of peripheral ghrelin expression under conditions of AN.

ABA mice displayed an increase of *preproghrelin* mRNA-expressing cells of the stomach in proportion to body weight loss [78]. Interestingly, hypothalamic *preproghrelin* mRNA expression was suppressed in ABA mice [78], possibly pointing toward differential effects of central and peripheral ghrelin.

It has been reported that under conditions of food restriction in rats oxidative soleus type muscles are more susceptible to circulating ghrelin compared to those of the glycolytic gastrocnemius type [79], giving rise to a differential regulation of the ghrelin receptor due to anorexia, as was reported for e.g., hypothyroidism [80], a hypothesis to be further investigated.

### 2.3. Acyl and Desacyl Ghrelin

Subsequent studies investigated different forms of ghrelin showing that acyl ghrelin was significantly increased in AN [81,82], likely to stimulate food intake in a compensatory fashion. It is to note that another group described opposing results: an increase of desacyl ghrelin in AN and a negative correlation with BMI, while acyl ghrelin was not/only slightly different without reaching significance compared to healthy controls [83,84]. Adding more confusion, a study reported both, acyl and desacyl ghrelin to be increased in patients with AN-R [85], whereas in constitutionally lean individuals acyl ghrelin was observed to be decreased compared to normal weight controls [86]. These differences might be due to different BMI values of the anorexic subjects studied (13.9 ± 1.0 [81] vs. 15.5 ± 2.6 [84] vs. 13.4 ± 0.3 [85]) or population size (*n* = 5 [81] vs. *n* = 30 [84] vs. *n* = 10 [85]). Observing a circadian profile, patients with AN-R showed higher total and acyl ghrelin levels compared to controls, whereas subjects with AN-BP displayed decreased total and acyl ghrelin levels; moreover, the ratio of acyl/total ghrelin was decreased in AN-BP [87,88]. The incongruence to findings mentioned before presenting highest levels in AN-BP, followed by high levels in AN-R and the lowest levels of ghrelin in healthy controls [65] could be explained by the different time points/period of time of blood sampling as well as by the different BMI values (15.4 ± 1.4 and 15.2 ± 1.6 in [87] vs. 13.7 ± 1.9 and 13.6 ± 1.5 in [65]).

ELISA (enzyme-linked immunosorbent assay) examinations showed decreased auto-antibodies, namely immune globulin G (IgG), IgM and IgA against acyl ghrelin and elevated levels of IgG auto-antibodies against desacyl ghrelin in AN but not healthy subjects [89]. These auto-antibodies against desacyl ghrelin were found mostly in immune complexes with desacyl ghrelin and were negatively associated with plasma acyl and desacyl ghrelin, whereas acyl ghrelin IgM auto-antibodies correlated with BMI [89]. It might be hypothesized that IgG against acyl ghrelin are reduced to allow free acyl ghrelin to stimulate food intake, whereas desacyl ghrelin’s effect to lower food intake might be inhibited by IgG bound to desacyl ghrelin leading to immune complexes in AN. In infants with Prader-Willi syndrome elevated levels of desacyl ghrelin were observed, which have been implicated in the anorexia observed at the beginning of the disease [90], giving rise to desacyl ghrelin’s inhibition as a possible target in the treatment of anorexia (nervosa).

### 2.4. Nutrient-Related Alterations of Ghrelin

Physiologically, ghrelin is elevated before a meal and decreases after food consumption. However, unlike in control subjects, acyl ghrelin levels in plasma from anorexic women did not decrease after meal consumption [91]. Other studies observed a moderate (−25%) reduction of acyl ghrelin with a delayed onset (60–90 min after the meal) in subjects with AN following a mixed meal [64] or a high-carbohydrate breakfast [92] which may represent a compensatory action to promote further food intake. It is to note that especially fat intake predicted ghrelin values in plasma of patients with AN [93], likely related to the need of fatty acids for ghrelin’s acylation in the stomach [24]. This is further supported by the finding that consumption of medium-chain triglycerides elevates ghrelin levels by activation of GOAT [94].

In contrast to food intake, modified sham feeding, where food is smelled and chewed but not swallowed, resulted in an elevation of circulating ghrelin concentrations which was higher in AN compared to age-matched healthy women [69], pointing towards an increased vagal tone in AN, whose hyperactivity was described in adolescents suffering from AN before when investigating cardiac functions [95].

Interestingly, in AN subjects the consumption of both favorite and unfavorite foods resulted in the reduction of ghrelin levels while in healthy as well as former anorexic subjects eating of favorite food further increased ghrelin levels [96]. To investigate underlying mechanisms, functional magnetic resonance imaging can be used that indicated a positive relation between fasting acyl ghrelin and activity in the right amygdala, hippocampus, insula, orbitofrontal cortex in response to high caloric foods in normal weight subjects [97]. The association between acyl ghrelin and hippocampus activity following a visual stimulus showing high caloric food was absent in AN but restored in AN patients following weight recovery [97] indicating altered reward responsivity in AN.

During the oral glucose tolerance test (oGTT) subjects receive 75 mL of glucose solution which induced a reduction of circulating ghrelin levels in healthy subjects [98]. An early study reported that after an oGTT total ghrelin concentrations in plasma decreased both in AN (−49%) and healthy age-matched controls (−57%); likewise, mean levels of plasma acyl ghrelin decreased in both groups [81]. Interestingly, the type of AN had an impact on the kinetics of ghrelin changes with a 58-% reduction of total ghrelin after 120 min in AN-BP, while in AN-R the 80-% decline was observed 180 min after the oGTT [99]. The mechanisms underlying this different time course warrant further investigation. Another study did not observe a decrease of total ghrelin following glucose neither in AN patients nor in healthy controls, while acyl ghrelin was significantly reduced [82] resulting in a significantly reduced acyl/total ghrelin ratio [82,83,100]. Interestingly, early after the test (at 30 and 60 min) total [101], acyl and desacyl ghrelin [85] were increased.

In contrast to the ghrelin responses observed, less than one third of adolescent patients showed an adequate reduction of GH levels following an oGTT [101]. Since ghrelin levels did not predict the oGTT-induced response of GH [101] altered GHSR1a sensitivity might play a role, a hypothesis to be further investigated.

### 2.5. Treatment-Related Alterations of Ghrelin

Weight regain of 14% in patients undergoing psychotherapy was associated with a 25-% reduction of fasting plasma ghrelin levels [52], other studies even reported a normalization [102] or a decline of plasma ghrelin below levels of control subjects [103]. Subsequent studies investigated these alterations in more detail. Treatment encompassing cognitive behavioral therapy and nutritional therapy led to a decrease of ghrelin levels in patients with AN emergently hospitalized as well as in AN-BP patients, while in AN-R ghrelin levels even exceeded levels before beginning of treatment and control levels [68]. It is to note that AN-BP patients still had elevated ghrelin levels at the end of the treatment [68], likely due to the fact that they did not reach the desired weight gain. Another study reported that relative ghrelin changes did not differ before and after treatment, while absolute values of postprandial ghrelin decreased from admission, partial weight gain and discharge, respectively (871.9, 597.0, 570.4 pg/mL) [104]. These changes were also observed over a long period of time (24 weeks) [105] and could be reinstated after a short nutritional rehabilitation program [106].

Investigating the different forms of ghrelin, a study applying cognitive behavior therapy and nutritional rehabilitation described a decrease of desacyl ghrelin resulting in an increased acyl/total ghrelin ratio [88]. This decrease of desacyl ghrelin was also observed in another study [107] and might be accompanied by a reduction in anorexigenic signaling.

A study investigating acute meal-related changes observed that the postprandial decrease of ghrelin was similar in anorexic patients and healthy controls reaching the nadir at 120 min [108]. However, in AN, ghrelin levels remained higher in comparison to healthy controls even after therapy comprised of inpatient behavioral treatment involving operant techniques, nutritional rehabilitation, psychotherapy, family therapy, and behavioral counseling, application of serotonin reuptake inhibitors and sulpiride as well as nutritional therapy [108]. It is important to note that other multimodal treatment approaches—here including cognitive behavioral psychotherapy and programmed nutritional rehabilitation, combined with olanzapine or placebo—did not induce alterations of ghrelin in AN patients [109], likely associated with the severity of the disease at the beginning of the treatment (BMI 12.4 ± 1.7 vs. 16.3 ± 0.7).

## 3. Genetics Contributing to Altered Ghrelin Signaling in Anorexia Nervosa

Two studies in 2006 examined the association between several polymorphisms of the *ghrelin* gene including Gln90Leu, Leu72Met, Arg51Gln described in obese and healthy humans before [110,111] and the development of AN, observing no significant difference in the frequency of those gene variants among anorexic and healthy subjects in Caucasian women or a mixed European population from Italy, Spain, Germany, Slovenia, France, Austria and United Kingdom [112,113].

Subsequently, a significant correlation between the polymorphism Leu72Met, a region potentially responsible for posttranslational processing, and the prevalence of AN-BP was documented [114]. This polymorphism was predominantly detected in subjects with low BMI, fat mass and fasting respiratory quotient [114]. In addition, regarding the Gln90Leu72 haplotype a significant transmission misbalance in AN patients was detected, analyzing AN patients and both parents in a French population [114].

Regarding the recovery potential of anorexic patients, the TT genotype at 3056 T→C in the *ghrelin* gene showed higher probability to achieve normal body weight [115]. An above-average prevalence of the G/G genotype at the SNP (single nucleotide polymorphism) vs100096097 was discovered in German anorexic subjects, resulting in a genetic variation in the GOAT enzyme [116]. Since GOAT is reduced in patients with AN [21] and may thereby contribute to the reduced orexigenic drive in these patients, this genetic alteration may well play a role in the pathophysiology of the disease. These changes and a possible impact on treatment outcomes should be further evaluated in future studies.

## 4. Effects of Exogenous Ghrelin in Anorexia Nervosa

### 4.1. Effects of Ghrelin Administration

Due to the robust orexigenic effect of ghrelin, several studies investigated a potential therapeutic use of the peptide. In ABA mice a GHSR1a antagonist, injected either acutely intracerebroventricularly or chronically peripherally, inhibited food-anticipatory behavior but had no effect on total food intake [62]. Intraperitoneal injection of ghrelin led to a blunted reduction of food intake under conditions of ABA, preventing the development of ABA, nonetheless, animals still lost body weight [117]. When combined with plasmatic anti-ghrelin IgG from obese, which is able to protect ghrelin from degradation, thereby enhancing ghrelin’s orexigenic effects [118], ghrelin inhibited physical activity during feeding and stimulated the activity after meal consumption resulting in an elevated relation of consumed food to running distance during feeding time in ghrelin plus IgG-receiving animals [117].

Modulations of ghrelin signaling were also tested in humans. Intravenous injection of ghrelin only moderately increased GH, giving rise to a desensitization of the GHSR1a under conditions of AN [119]. However, ghrelin induced a regular increase of ACTH (adrenocorticotropic hormone) and cortisol in AN [119], possibly pointing towards the mediation by different receptor subtypes. Another study observed that ghrelin subjectively did not induce appetite but sleepiness in AN patients compared to constitutionally lean subjects [120], probably due to the ability of ghrelin to promote slow wave sleep in humans [121]. Moreover, acyl ghrelin infused intravenously over 5 h increased glucose levels in AN and even more in constitutionally lean subjects [122], possibly due to larger glycogen stores under constitutionally lean conditions.

Chronic intravenous application (twice a day over 14 days) of ghrelin at a dose of 3 ug/kg body weight resulted in improved epigastric discomfort and constipation in four of five patients, likely associated with increased gastric motility [123]. Also hunger scores were increased leading to an increased energy intake of 12% to 36% [123]. Similarly, in another study following the same administration protocol, ghrelin suppressed upper abdominal fullness inducing stomach rumble and hunger sensation but not constipation in five AN-R patients associated with an increase of daily energy intake by 20% [100]. Despite these encouraging results, these findings must be followed up in a larger sample under controlled conditions.

### 4.2. Effects of Ghrelin-Related Products and Ghrelin Receptor Agonists

Not only ghrelin itself displayed beneficial effects in the course of AN, but also the ghrelin enhancer rikkunshito, a traditional Japanese medicine, was shown in vitro to antagonize serotonin’s action in POMC (pro-opiomelanocortin) neurons of the arcuate nucleus, whose release has been described to be increased in AN [124]. Additionally, in cisplatin-treated rats rikkunshito was able to prevent the reduction of plasma ghrelin levels by inhibiting ghrelin’s desacylation, resulting in an augmented acyl/desacyl ghrelin ratio associated with a blunted decrease of food intake [125].

In humans, the effect of rikkunshito has been tested in chemotherapy-induced anorexia only, leading to a reduction of nausea and emesis and a stimulation of appetite [126]. These findings should be followed up in patients with AN as well. Very recently, a study investigated the effects of relamorelin (subcutaneously daily over a period of four weeks), a ghrelin agonist, in an outpatient cohort (*n* = 10–12/group) of AN showing a significant acceleration of gastric emptying as well as a trend towards an increase of body weight (+0.9 kg vs. 0.3 kg in the control group, *p* < 0.07) [127]. Again, larger follow up studies are needed. These findings are surprising as ghrelin levels are already elevated, and ghrelin resistance is suspected as described above. Nonetheless, the further increase of ghrelin to supraphysiological levels might still be a promising approach to stimulate food intake in a supportive manner.

## 5. Conclusions

**Figure 1:**
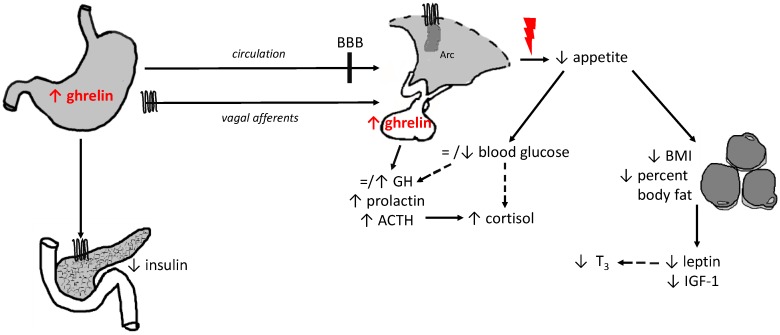
Hypothesized alterations of ghrelin’s signaling in anorexia nervosa. 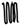 growth hormone secretagogue receptor 1a; 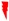 mechanisms contributing to insufficient stimulation of food intake irrespective of high ghrelin levels; = no alteration; ↑ increase/stimulation; ↓ decrease/inhibition, - - > indirect effect; ACTH, adrenocorticotropic hormone; BBB, brain-blood barrier; BMI, body mass index; GH, growth hormone; IGF-1, insulin-like growth factor-1; T_3_, triiodothyronine.

In conclusion, ghrelin is the only known peripherally produced and centrally acting peptide hormone that stimulates food intake and gastric motility. Additionally, ghrelin increases blood glucose levels by reducing glucose-stimulated insulin secretion, insulin sensitivity and inducing glucagon secretion [31]. In states of severe undernutrition and underweight such as AN, ghrelin is increased compared to healthy controls, even if those are BMI-matched [70], which can be normalized through weight gain and renutrition [102]. Genome-wide association studies demonstrated a relationship between polymorphisms in the ghrelin gene [114] as well as in the gene of the enzyme acylating ghrelin, GOAT [116] and the prevalence of AN [114], pointing towards a ghrelin-related genetic component of AN. In AN not only central [77] and peripheral [78] ghrelin expression is increased, but also ghrelin signaling and modulation are impaired as indicated by a delayed or absent postprandial decrease of ghrelin [64,91], the inability to suppress GH secretion adequately after glucose digestion [101] or the insufficiency of glucose elevation after exogenous ghrelin application [119], suggesting ghrelin resistance in AN (Figure 1). Noteworthy, exogenous ghrelin (by raising ghrelin to supraphysiological levels) or ghrelin receptor agonists still might be able to improve the course of AN by stimulating appetite and reducing gastric discomfort leading to an increase of energy intake and body weight [100,123,127]. Since these data are derived from small pilot studies, these effects should be corroborated—or refuted—in larger clinical trials.

## Abbreviations

GHSR	growth hormone secretagogue receptor
AgRP	agouti-related peptide
NPY	neuropeptide Y
GOAT	ghrelin-*O*-acyl transferase
GIP	gastric inhibitory polypeptide/glucose-dependent insulinotropic peptide
mRNA	messenger ribonucleic acid
mTORC1/S6K1	mechanistic target of rapamycin complex 1/p70 ribosomal protein kinase 1
AN	anorexia nervosa
BMI	body mass index
ABA	activity-based anorexia
AN-BP	anorexia nervosa of bingeing/purging subtype
AN-R	anorexia nervosa of restrictive subtype
BULIT-R	bulimia test—DSM-III-R
GH	growth hormone
IGF	insulin-like growth factor
HOMA-IR	homeostatic model assessment—insulin resistance
T_3_	triiodothyronine
ELISA	enzyme-linked immunosorbent assay
Ig	immune globulin
IGF-1	insulin-like growth factor 1
oGTT	oral glucose tolerance test
SNP	single nucleotide polymorphism
ACTH	adrenocorticotropic hormone
POMC	pro-opiomelanocortin

## References

[1] Kojima M., Hosoda H., Date Y., Nakazato M., Matsuo H., Kangawa K. (1999). Ghrelin is a growth-hormone-releasing acylated peptide from stomach. Nature.

[2] Warzecha Z., Dembiński A., Ceranowicz P., Dembiński M., Cieszkowski J., Konturek S.J., Polus A., Pawlik W.W., Kuwahara A., Kato I. (2006). Influence of ghrelin on gastric and duodenal growth and expression of digestive enzymes in young mature rats. J. Physiol. Pharmacol..

[3] Willesen M.G., Kristensen P., Rømer J. (1999). Co-localization of growth hormone secretagogue receptor and NPY mRNA in the arcuate nucleus of the rat. Neuroendocrinology.

[4] Tschöp M., Smiley D.L., Heiman M.L. (2000). Ghrelin induces adiposity in rodents. Nature.

[5] Yang J., Brown M.S., Liang G., Grishin N.V., Goldstein J.L. (2008). Identification of the acyltransferase that octanoylates ghrelin, an appetite-stimulating peptide hormone. Cell.

[6] Zhang J.V., Ren P.G., Avsian-Kretchmer O., Luo C.W., Rauch R., Klein C., Hsueh A.J. (2005). Obestatin, a peptide encoded by the ghrelin gene, opposes ghrelin’s effects on food intake. Science.

[7] Goebel-Stengel M., Stengel A., Taché Y. (2008). Continued controversy on obestatin as a gut hormone influencing food intake and gastrointestinal motility. Obes. Metab..

[8] Druce M.R., Wren A.M., Park A.J., Milton J.E., Patterson M., Frost G., Ghatei M.A., Small C., Bloom S.R. (2005). Ghrelin increases food intake in obese as well as lean subjects. Int. J. Obes..

[9] Broglio F., Arvat E., Benso A., Gottero C., Muccioli G., Papotti M., van der Lely A.J., Deghenghi R., Ghigo E. (2001). Ghrelin, a natural GH secretagogue produced by the stomach, induces hyperglycemia and reduces insulin secretion in humans. J. Clin. Endocrinol. Metab..

[10] Tack J., Depoortere I., Bisschops R., Delporte C., Coulie B., Meulemans A., Janssens J., Peeters T. (2006). Influence of ghrelin on interdigestive gastrointestinal motility in humans. Gut.

[11] Inhoff T., Mönnikes H., Noetzel S., Stengel A., Goebel M., Dinh Q.T., Riedl A., Bannert N., Wisser A.S., Wiedenmann B. (2008). Desacyl ghrelin inhibits the orexigenic effect of peripherally injected ghrelin in rats. Peptides.

[12] Fernandez G., Cabral A., Cornejo M.P., De Francesco P.N., Garcia-Romero G., Reynaldo M., Perello M. (2016). Des-acyl ghrelin directly targets the arcuate nucleus in a ghrelin-receptor independent manner and impairs the orexigenic effect of ghrelin. J. Neuroendocrinol..

[13] Heppner K.M., Piechowski C.L., Müller A., Ottaway N., Sisley S., Smiley D.L., Habegger K.M., Pfluger P.T., Dimarchi R., Biebermann H. (2014). Both acyl and des-acyl ghrelin regulate adiposity and glucose metabolism via central nervous system ghrelin receptors. Diabetes.

[14] Gauna C., van de Zande B., van Kerkwijk A., Themmen A.P., van der Lely A.J., Delhanty P.J. (2007). Unacylated ghrelin is not a functional antagonist but a full agonist of the type 1a growth hormone secretagogue receptor (GHS-R). Mol. Cell. Endocrinol..

[15] Asakawa A., Inui A., Fujimiya M., Sakamaki R., Shinfuku N., Ueta Y., Meguid M.M., Kasuga M. (2005). Stomach regulates energy balance via acylated ghrelin and desacyl ghrelin. Gut.

[16] Ariyasu H., Takaya K., Tagami T., Ogawa Y., Hosoda K., Akamizu T., Suda M., Koh T., Natsui K., Toyooka S. (2001). Stomach is a major source of circulating ghrelin, and feeding state determines plasma ghrelin-like immunoreactivity levels in humans. J. Clin. Endocrinol. Metab..

[17] Date Y., Nakazato M., Hashiguchi S., Dezaki K., Mondal M.S., Hosoda H., Kojima M., Kangawa K., Arima T., Matsuo H. (2002). Ghrelin is present in pancreatic alpha-cells of humans and rats and stimulates insulin secretion. Diabetes.

[18] Gnanapavan S., Kola B., Bustin S.A., Morris D.G., McGee P., Fairclough P., Bhattacharya S., Carpenter R., Grossman A.B., Korbonits M. (2002). The tissue distribution of the mRNA of ghrelin and subtypes of its receptor, GHS-R, in humans. J. Clin. Endocrinol. Metab..

[19] Lim C.T., Kola B., Grossman A., Korbonits M. (2011). The expression of ghrelin o-acyltransferase (GOAT) in human tissues. Endocr. J..

[20] Stengel A., Goebel M., Wang L., Taché Y., Sachs G., Lambrecht N.W. (2010). Differential distribution of ghrelin-o-acyltransferase (GOAT) immunoreactive cells in the mouse and rat gastric oxyntic mucosa. Biochem. Biophys. Res. Commun..

[21] Goebel-Stengel M., Hofmann T., Elbelt U., Teuffel P., Ahnis A., Kobelt P., Lambrecht N.W., Klapp B.F., Stengel A. (2013). The ghrelin activating enzyme ghrelin-o-acyltransferase (GOAT) is present in human plasma and expressed dependent on body mass index. Peptides.

[22] Gutierrez J.A., Solenberg P.J., Perkins D.R., Willency J.A., Knierman M.D., Jin Z., Witcher D.R., Luo S., Onyia J.E., Hale J.E. (2008). Ghrelin octanoylation mediated by an orphan lipid transferase. Proc. Natl. Acad. Sci. USA.

[23] González C.R., Vázquez M.J., López M., Diéguez C. (2008). Influence of chronic undernutrition and leptin on GOAT mRNA levels in rat stomach mucosa. J. Mol. Endocrinol..

[24] Sakata I., Nakamura K., Yamazaki M., Matsubara M., Hayashi Y., Kangawa K., Sakai T. (2002). Ghrelin-producing cells exist as two types of cells, closed- and opened-type cells, in the rat gastrointestinal tract. Peptides.

[25] Iwakura H., Ariyasu H., Hosoda H., Yamada G., Hosoda K., Nakao K., Kangawa K., Akamizu T. (2011). Oxytocin and dopamine stimulate ghrelin secretion by the ghrelin-producing cell line, MGN3-1 in vitro. Endocrinology.

[26] Engelstoft M.S., Park W.M., Sakata I., Kristensen L.V., Husted A.S., Osborne-Lawrence S., Piper P.K., Walker A.K., Pedersen M.H., Nøhr M.K. (2013). Seven transmembrane G protein-coupled receptor repertoire of gastric ghrelin cells. Mol. Metab..

[27] Janssen S., Laermans J., Verhulst P.J., Thijs T., Tack J., Depoortere I. (2011). Bitter taste receptors and alpha gustducin regulate the secretion of ghrelin with functional effects on food intake and gastric emptying. Proc. Natl. Acad. Sci. USA..

[28] Shimada M., Date Y., Mondal M.S., Toshinai K., Shimbara T., Fukunaga K., Murakami N., Miyazato M., Kangawa K., Yoshimatsu H. (2003). Somatostatin suppresses ghrelin secretion from the rat stomach. Biochem. Biophys. Res. Commun..

[29] Stengel A., Goebel-Stengel M., Wang L., Shaikh A., Lambrecht N.W., Rivier J., Taché Y. (2011). Abdominal surgery inhibits circulating acyl ghrelin and ghrelin-O-acyltransferase levels in rats: Role of the somatostatin receptor subtype 2. Am. J. Physiol. Gastrointest. Liver Physiol..

[30] Ceranowicz P., Warzecha Z., Dembiński A. (2015). Peptidyl hormones of endocrine cells origin in the gut—Their discovery and physiological relevance. J. Physiol. Pharmacol..

[31] Weibert E., Stengel A. (2017). The X/A-like cell revisited—Spotlight on the peripheral effects of NUCB2/nesfatin-1 and ghrelin. J. Physiol. Pharmacol..

[32] Zhang Q., Huang C., Meng B., Tang T., Shi Q., Yang H. (2012). Acute effect of ghrelin on ischemia/reperfusion injury in the rat spinal cord. Int. J. Mol. Sci..

[33] Frascarelli S., Ghelardoni S., Ronca-Testoni S., Zucchi R. (2003). Effect of ghrelin and synthetic growth hormone secretagogues in normal and ischemic rat heart. Basic Res. Cardiol..

[34] Takeda R., Nishimatsu H., Suzuki E., Satonaka H., Nagata D., Oba S., Sata M., Takahashi M., Yamamoto Y., Terauchi Y. (2006). Ghrelin improves renal function in mice with ischemic acute renal failure. J. Am. Soc. Nephrol..

[35] Warzecha Z., Ceranowicz P., Dembiński A., Cieszkowski J., Kuśnierz-Cabala B., Tomaszewska R., Kuwahara A., Kato I. (2010). Therapeutic effect of ghrelin in the course of cerulein-induced acute pancreatitis in rats. J. Physiol. Pharmacol..

[36] Ceranowicz D., Warzecha Z., Dembiński A., Ceranowicz P., Cieszkowski J., Kuśnierz-Cabala B., Tomaszewska R., Kuwahara A., Kato I. (2010). Role of hormonal axis, growth hormone—IGF-1, in the therapeutic effect of ghrelin in the course of cerulein-induced acute pancreatitis. J. Physiol. Pharmacol..

[37] Bukowczan J., Warzecha Z., Ceranowicz P., Kuśnierz-Cabala B., Tomaszewska R., Dembiński A. (2015). Therapeutic effect of ghrelin in the course of ischemia/reperfusion-induced acute pancreatitis. Curr. Pharm. Des..

[38] Dembiński A., Warzecha Z., Ceranowicz P., Cieszkowski J., Pawlik W.W., Tomaszewska R., Kuśnierz-Cabala B., Naskalski J.W., Kuwahara A., Kato I. (2006). Role of growth hormone and insulin-like growth factor-1 in the protective effect of ghrelin in ischemia/reperfusion-induced acute pancreatitis. Growth Horm. IGF Res..

[39] Cieszkowski J., Warzecha Z., Ceranowicz P., Ceranowicz D., Kusnierz-Cabala B., Pedziwiatr M., Dembiński M., Ambrozy T., Kaczmarzyk T., Pihut M. (2017). Therapeutic effect of exogenous ghrelin in the healing of gingival ulcers is mediated by the release of endogenous growth hormone and insulin-like growth factor-1. J. Physiol. Pharmacol..

[40] Warzecha Z., Kownacki P., Ceranowicz P., Dembiński M., Cieszkowski J., Dembiński A. (2013). Ghrelin accelerates the healing of oral ulcers in non-sialoadenectomized and sialoadenectomized rats. J. Physiol. Pharmacol..

[41] Warzecha Z., Ceranowicz D., Dembiński A., Ceranowicz P., Cieszkowski J., Kuwahara A., Kato I., Dembinski M., Konturek P.C. (2012). Ghrelin accelerates the healing of cysteamine-induced duodenal ulcers in rats. Med. Sci. Monit..

[42] Ceranowicz P., Warzecha Z., Dembinski A., Sendur R., Cieszkowski J., Ceranowicz D., Pawlik W.W., Kuwahara A., Kato I., Konturek P.C. (2009). Treatment with ghrelin accelerates the healing of acetic acid-induced gastric and duodenal ulcers in rats. J. Physiol. Pharmacol..

[43] Matuszyk A., Ceranowicz P., Warzecha Z., Cieszkowski J., Ceranowicz D., Gałązka K., Bonior J., Jaworek J., Bartuś K., Gil K. (2016). Exogenous ghrelin accelerates the healing of acetic acid-induced colitis in rats. Int. J. Mol. Sci..

[44] Maduzia D., Matuszyk A., Ceranowicz D., Warzecha Z., Ceranowicz P., Fyderek K., Gałązka K., Dembiński A. (2015). The influence of pretreatment with ghrelin on the development of acetic-acid-induced colitis in rats. J. Physiol. Pharmacol..

[45] Matuszyk A., Ceranowicz D., Warzecha Z., Ceranowicz P., Fyderek K., Gałązka K., Cieszkowski J., Bonior J., Jaworek J., Pihut M. (2015). The influence of ghrelin on the development of dextran sodium sulfate-induced colitis in rats. BioMed. Res. Int..

[46] Cummings D.E., Purnell J.Q., Frayo R.S., Schmidova K., Wisse B.E., Weigle D.S. (2001). A preprandial rise in plasma ghrelin levels suggests a role in meal initiation in humans. Diabetes.

[47] Tschöp M., Wawarta R., Riepl R.L., Friedrich S., Bidlingmaier M., Landgraf R., Folwaczny C. (2001). Post-prandial decrease of circulating human ghrelin levels. J. Endocrinol. Investig..

[48] Monteleone P., Bencivenga R., Longobardi N., Serritella C., Maj M. (2003). Differential responses of circulating ghrelin to high-fat or high-carbohydrate meal in healthy women. J. Clin. Endocrinol. Metab..

[49] Mizutani M., Atsuchi K., Asakawa A., Matsuda N., Fujimura M., Inui A., Kato I., Fujimiya M. (2009). Localization of acyl ghrelin- and des-acyl ghrelin-immunoreactive cells in the rat stomach and their responses to intragastric pH. Am. J. Physiol. Gastrointest. Liver Physiol..

[50] Tschöp M., Weyer C., Tataranni P.A., Devanarayan V., Ravussin E., Heiman M.L. (2001). Circulating ghrelin levels are decreased in human obesity. Diabetes.

[51] Garcia J.M., Garcia-Touza M., Hijazi R.A., Taffet G., Epner D., Mann D., Smith R.G., Cunningham G.R., Marcelli M. (2005). Active ghrelin levels and active to total ghrelin ratio in cancer-induced cachexia. J. Clin. Endocrinol. Metab..

[52] Otto B., Cuntz U., Fruehauf E., Wawarta R., Folwaczny C., Riepl R.L., Heiman M.L., Lehnert P., Fichter M., Tschöp M. (2001). Weight gain decreases elevated plasma ghrelin concentrations of patients with anorexia nervosa. Eur. J. Endocrinol..

[53] Nakazato M., Murakami N., Date Y., Kojima M., Matsuo H., Kangawa K., Matsukura S. (2001). A role for ghrelin in the central regulation of feeding. Nature.

[54] Stevanovic D., Trajkovic V., Müller-Lühlhoff S., Brandt E., Abplanalp W., Bumke-Vogt C., Liehl B., Wiedmer P., Janjetovic K., Starcevic V. (2013). Ghrelin-induced food intake and adiposity depend on central mTORC1/S6K1 signaling. Mol. Cell. Endocrinol..

[55] Date Y., Murakami N., Toshinai K., Matsukura S., Niijima A., Matsuo H., Kangawa K., Nakazato M. (2002). The role of the gastric afferent vagal nerve in ghrelin-induced feeding and growth hormone secretion in rats. Gastroenterology.

[56] Zipfel S., Giel K.E., Bulik C.M., Hay P., Schmidt U. (2015). Anorexia nervosa: Aetiology, assessment, and treatment. Lancet Psychiatry.

[57] Sim L.A., McAlpine D.E., Grothe K.B., Himes S.M., Cockerill R.G., Clark M.M. (2010). Identification and treatment of eating disorders in the primary care setting. Mayo Clin. Proc..

[58] Lindvall Dahlgren C., Wisting L., Rø Ø. (2017). Feeding and eating disorders in the DSM-5 era: A systematic review of prevalence rates in non-clinical male and female samples. J. Eat. Disord..

[59] Shiiya T., Nakazato M., Mizuta M., Date Y., Mondal M.S., Tanaka M., Nozoe S., Hosoda H., Kangawa K., Matsukura S. (2002). Plasma ghrelin levels in lean and obese humans and the effect of glucose on ghrelin secretion. J. Clin. Endocrinol. Metab..

[60] Rigamonti A.E., Pincelli A.I., Corra B., Viarengo R., Bonomo S.M., Galimberti D., Scacchi M., Scarpini E., Cavagnini F., Müller E.E. (2002). Plasma ghrelin concentrations in elderly subjects: Comparison with anorexic and obese patients. J. Endocrinol..

[61] Méquinion M., Caron E., Zgheib S., Stievenard A., Zizzari P., Tolle V., Cortet B., Lucas S., Prévot V., Chauveau C. (2015). Physical activity: Benefit or weakness in metabolic adaptations in a mouse model of chronic food restriction?. Am. J. Physiol. Endocrinol. Metab..

[62] Verhagen L.A., Egecioglu E., Luijendijk M.C., Hillebrand J.J., Adan R.A., Dickson S.L. (2011). Acute and chronic suppression of the central ghrelin signaling system reveals a role in food anticipatory activity. Eur. Neuropsychopharmacol..

[63] Hofmann T., Elbelt U., Haas V., Ahnis A., Klapp B.F., Rose M., Stengel A. (2017). Plasma kisspeptin and ghrelin levels are independently correlated with physical activity in patients with anorexia nervosa. Appetite.

[64] Stock S., Leichner P., Wong A.C., Ghatei M.A., Kieffer T.J., Bloom S.R., Chanoine J.P. (2005). Ghrelin, peptide YY, glucose-dependent insulinotropic polypeptide, and hunger responses to a mixed meal in anorexic, obese, and control female adolescents. J. Clin. Endocrinol. Metab..

[65] Tanaka M., Naruo T., Yasuhara D., Tatebe Y., Nagai N., Shiiya T., Nakazato M., Matsukura S., Nozoe S. (2003). Fasting plasma ghrelin levels in subtypes of anorexia nervosa. Psychoneuroendocrinology.

[66] Tanaka M., Naruo T., Nagai N., Kuroki N., Shiiya T., Nakazato M., Matsukura S., Nozoe S. (2003). Habitual binge/purge behavior influences circulating ghrelin levels in eating disorders. J. Psychiatr. Res..

[67] Troisi A., Di Lorenzo G., Lega I., Tesauro M., Bertoli A., Leo R., Iantorno M., Pecchioli C., Rizza S., Turriziani M. (2005). Plasma ghrelin in anorexia, bulimia, and binge-eating disorder: Relations with eating patterns and circulating concentrations of cortisol and thyroid hormones. Neuroendocrinology.

[68] Tanaka M., Nakahara T., Kojima S., Nakano T., Muranaga T., Nagai N., Ueno H., Nakazato M., Nozoe S., Naruo T. (2004). Effect of nutritional rehabilitation on circulating ghrelin and growth hormone levels in patients with anorexia nervosa. Regul. Pept..

[69] Monteleone P., Serritella C., Martiadis V., Maj M. (2008). Deranged secretion of ghrelin and obestatin in the cephalic phase of vagal stimulation in women with anorexia nervosa. Biol. Psychiatry.

[70] Tolle V., Kadem M., Bluet-Pajot M.T., Frere D., Foulon C., Bossu C., Dardennes R., Mounier C., Zizzari P., Lang F. (2003). Balance in ghrelin and leptin plasma levels in anorexia nervosa patients and constitutionally thin women. J. Clin. Endocrinol. Metab..

[71] Germain N., Galusca B., Le Roux C.W., Bossu C., Ghatei M.A., Lang F., Bloom S.R., Estour B. (2007). Constitutional thinness and lean anorexia nervosa display opposite concentrations of peptide YY, glucagon-like peptide 1, ghrelin, and leptin. Am. J. Clin. Nutr..

[72] Blauwhoff-Buskermolen S., Langius J.A., Heijboer A.C., Becker A., de van der Schueren M.A., Verheul H.M. (2017). Plasma ghrelin levels are associated with anorexia but not cachexia in patients with NSCLC. Front. Physiol..

[73] Misra M., Miller K.K., Kuo K., Griffin K., Stewart V., Hunter E., Herzog D.B., Klibanski A. (2005). Secretory dynamics of ghrelin in adolescent girls with anorexia nervosa and healthy adolescents. Am. J. Physiol. Endocrinol. Metab..

[74] Takaya J., Hattori Y., Ishizaki Y., Kaneko K. (2008). Surged leptin/ghrelin secretion associated with anorexia nervosa. J. Pediatr. Gastroenterol. Nutr..

[75] Hawkes C.P., Grimberg A. (2015). Insulin-like growth factor-I is a marker for the nutritional state. Pediatr. Endocrinol. Rev..

[76] Baumgard L.H., Hausman G.J., Sanz Fernandez M.V. (2016). Insulin: Pancreatic secretion and adipocyte regulation. Domest. Anim. Endocrinol..

[77] Rotondo F., Scheithauer B.W., Syro L.V., Rotondo A., Kovacs K. (2012). Pituitary immunoexpression of ghrelin in anorexia nervosa. Pituitary.

[78] François M., Barde S., Achamrah N., Breton J., do Rego J.C., Coëffier M., Hökfelt T., Déchelotte P., Fetissov S.O. (2015). The number of preproghrelin mRNA expressing cells is increased in mice with activity-based anorexia. Neuropeptides.

[79] Pardo M., Roca-Rivada A., Al-Massadi O., Seoane L.M., Camiña J.P., Casanueva F.F. (2010). Peripheral leptin and ghrelin receptors are regulated in a tissue-specific manner in activity-based anorexia. Peptides.

[80] Edwards C.A., Dieguez C., Scanlon M.F. (1989). Effects of hypothyroidism, tri-iodothyronine and glucocorticoids on growth hormone responses to growth hormone-releasing hormone and His-D-Trp-Ala-Trp-D-Phe-Lys-NH2. J. Endocrinol..

[81] Nakai Y., Hosoda H., Nin K., Ooya C., Hayashi H., Akamizu T., Kangawa K. (2003). Plasma levels of active form of ghrelin during oral glucose tolerance test in patients with anorexia nervosa. Eur. J. Endocrinol..

[82] Nakai Y., Hosoda H., Nin K., Ooya C., Hayashi H., Akamizu T., Kangawa K. (2004). Short-term secretory regulation of the active form of ghrelin and total ghrelin during an oral glucose tolerance test in patients with anorexia nervosa. Eur. J. Endocrinol..

[83] Hotta M., Ohwada R., Katakami H., Shibasaki T., Hizuka N., Takano K. (2004). Plasma levels of intact and degraded ghrelin and their responses to glucose infusion in anorexia nervosa. J. Clin. Endocrinol. Metab..

[84] Kawai K., Yamanaka T., Yamashita S., Gondo M., Morita C., Arimura C., Nozaki T., Takii M., Kubo C. (2008). Somatic and psychological factors related to the body mass index of patients with anorexia nervosa. Eat. Weight Disord..

[85] Harada T., Nakahara T., Yasuhara D., Kojima S., Sagiyama K., Amitani H., Laviano A., Naruo T., Inui A. (2008). Obestatin, acyl ghrelin, and des-acyl ghrelin responses to an oral glucose tolerance test in the restricting type of anorexia nervosa. Biol. Psychiatry.

[86] Germain N., Galusca B., Grouselle D., Frere D., Tolle V., Zizzari P., Lang F., Epelbaum J., Estour B. (2009). Ghrelin/obestatin ratio in two populations with low body weight: Constitutional thinness and anorexia nervosa. Psychoneuroendocrinology.

[87] Germain N., Galusca B., Grouselle D., Frere D., Billard S., Epelbaum J., Estour B. (2010). Ghrelin and obestatin circadian levels differentiate bingeing-purging from restrictive anorexia nervosa. J. Clin. Endocrinol. Metab..

[88] Koyama K.I., Yasuhara D., Nakahara T., Harada T., Uehara M., Ushikai M., Asakawa A., Inui A. (2010). Changes in acyl ghrelin, des-acyl ghrelin, and ratio of acyl ghrelin to total ghrelin with short-term refeeding in female inpatients with restricting-type anorexia nervosa. Horm. Metab. Res..

[89] Terashi M., Asakawa A., Harada T., Ushikai M., Coquerel Q., Sinno M.H., Déchelotte P., Inui A., Fetissov S.O. (2011). Ghrelin reactive autoantibodies in restrictive anorexia nervosa. Nutrition.

[90] Beauloye V., Diene G., Kuppens R., Zech F., Winandy C., Molinas C., Faye S., Kieffer I., Beckers D., Nergardh R. (2016). High unacylated ghrelin levels support the concept of anorexia in infants with prader-willi syndrome. Orphanet J. Rare Dis..

[91] Nedvídková J., Krykorková I., Barták V., Papezová H., Gold P.W., Alesci S., Pacak K. (2003). Loss of meal-induced decrease in plasma ghrelin levels in patients with anorexia nervosa. J. Clin. Endocrinol. Metab..

[92] Sedláčkova D., Kopečková J., Papežová H., Vybíral S., Kvasnčcková H., Hill M., Nedvídková J. (2011). Changes of plasma obestatin, ghrelin and NPY in anorexia and bulimia nervosa patients before and after a high-carbohydrate breakfast. Physiol. Res..

[93] Misra M., Tsai P., Anderson E.J., Hubbard J.L., Gallagher K., Soyka L.A., Miller K.K., Herzog D.B., Klibanski A. (2006). Nutrient intake in community-dwelling adolescent girls with anorexia nervosa and in healthy adolescents. Am. J. Clin. Nutr..

[94] Kawai K., Nakashima M., Kojima M., Yamashita S., Takakura S., Shimizu M., Kubo C., Sudo N. (2017). Ghrelin activation and neuropeptide Y elevation in response to medium chain triglyceride administration in anorexia nervosa patients. Clin. Nutr. ESPEN.

[95] Kollai M., Bonyhay I., Jokkel G., Szonyi L. (1994). Cardiac vagal hyperactivity in adolescent anorexia nervosa. Eur. Heart J..

[96] Maria Monteleone A., Monteleone P., Dalle Grave R., Nigro M., El Ghoch M., Calugi S., Cimino M., Maj M. (2016). Ghrelin response to hedonic eating in underweight and short-term weight restored patients with anorexia nervosa. Psychiatry Res..

[97] Holsen L.M., Lawson E.A., Christensen K., Klibanski A., Goldstein J.M. (2014). Abnormal relationships between the neural response to high- and low-calorie foods and endogenous acylated ghrelin in women with active and weight-recovered anorexia nervosa. Psychiatry Res..

[98] Paslakis G., Westphal S., Hamann B., Gilles M., Lederbogen F., Deuschle M. (2014). Unstimulated and glucose-stimulated ghrelin in depressed patients and controls. J. Psychopharmacol..

[99] Tanaka M., Tatebe Y., Nakahara T., Yasuhara D., Sagiyama K., Muranaga T., Ueno H., Nakazato M., Nozoe S., Naruo T. (2003). Eating pattern and the effect of oral glucose on ghrelin and insulin secretion in patients with anorexia nervosa. Clin. Endocrinol. (Oxf.).

[100] Hotta M., Ohwada R., Akamizu T., Shibasaki T., Kangawa K. (2012). Therapeutic potential of ghrelin in restricting-type anorexia nervosa. Methods Enzymol..

[101] Misra M., Miller K.K., Herzog D.B., Ramaswamy K., Aggarwal A., Almazan C., Neubauer G., Breu J., Klibanski A. (2004). Growth hormone and ghrelin responses to an oral glucose load in adolescent girls with anorexia nervosa and controls. J. Clin. Endocrinol. Metab..

[102] Soriano-Guillén L., Barrios V., Campos-Barros A., Argente J. (2004). Ghrelin levels in obesity and anorexia nervosa: Effect of weight reduction or recuperation. J. Pediatr..

[103] Janas-Kozik M., Krupka-Matuszczyk I., Malinowska-Kolodziej I., Lewin-Kowalik J. (2007). Total ghrelin plasma level in patients with the restrictive type of anorexia nervosa. Regul. Pept..

[104] Otto B., Tschöp M., Frühauf E., Heldwein W., Fichter M., Otto C., Cuntz U. (2005). Postprandial ghrelin release in anorectic patients before and after weight gain. Psychoneuroendocrinology.

[105] Janas-Kozik M., Krupka-Matuszczyk I., Tomasik-Krótki J. (2006). Total plasma ghrelin level in anorexia nervosa female. Wiad. Lek..

[106] Beranová L., Sedlácková D., Kopecková J., Hainer V., Papezová H., Kvasnicková H., Nedvídková J. (2009). Neuropeptide Y, ghrelin and leptin plasma levels in anorexia nervosa patients and their changes during six-week refeeding. Vnitr. Lek..

[107] Uehara M., Yasuhara D., Nakahara T., Harada T., Koyama K.I., Ushikai M., Asakawa A., Inui A. (2011). Increase in energy intake leads to a decrease in obestatin in restricting-type of anorexia nervosa. Exp. Clin. Endocrinol. Diabetes.

[108] Nakahara T., Kojima S., Tanaka M., Yasuhara D., Harada T., Sagiyama K., Muranaga T., Nagai N., Nakazato M., Nozoe S. (2007). Incomplete restoration of the secretion of ghrelin and PYY compared to insulin after food ingestion following weight gain in anorexia nervosa. J. Psychiatr. Res..

[109] Brambilla F., Monteleone P., Maj M. (2007). Olanzapine-induced weight gain in anorexia nervosa: Iof leptin and ghrelin secretion?. Psychoneuroendocrinology.

[110] Ukkola O., Ravussin E., Jacobson P., Snyder E.E., Chagnon M., Sjöström L., Bouchard C. (2001). Mutations in the preproghrelin/ghrelin gene associated with obesity in humans. J. Clin. Endocrinol. Metab..

[111] Hinney A., Hoch A., Geller F., Schäfer H., Siegfried W., Goldschmidt H., Remschmidt H., Hebebrand J. (2002). Ghrelin gene: Identification of missense variants and a frameshift mutation in extremely obese children and adolescents and healthy normal weight students. J. Clin. Endocrinol. Metab..

[112] Monteleone P., Tortorella A., Castaldo E., Di Filippo C., Maj M. (2006). No association of the Arg51Gln and Leu72Met polymorphisms of the ghrelin gene with anorexia nervosa or bulimia nervosa. Neurosci. Lett..

[113] Cellini E., Nacmias B., Brecelj-Anderluh M., Badía-Casanovas A., Bellodi L., Boni C., Di Bella D., Estivill X., Fernandez-Aranda F., Foulon C. (2006). Case-control and combined family trios analysis of three polymorphisms in the ghrelin gene in european patients with anorexia and bulimia nervosa. Psychiatr. Genet..

[114] Dardennes R.M., Zizzari P., Tolle V., Foulon C., Kipman A., Romo L., Iancu-Gontard D., Boni C., Sinet P.M., Thérèse Bluet M. (2007). Family trios analysis of common polymorphisms in the obestatin/ghrelin, BDNF and AGRP genes in patients with anorexia nervosa: Association with subtype, body-mass index, severity and age of onset. Psychoneuroendocrinology.

[115] Ando T., Komaki G., Nishimura H., Naruo T., Okabe K., Kawai K., Takii M., Oka T., Kodama N., Nakamoto C. (2010). A ghrelin gene variant may predict crossover rate from restricting-type anorexia nervosa to other phenotypes of eating disorders: A retrospective survival analysis. Psychiatr. Genet..

[116] Müller T.D., Tschöp M.H., Jarick I., Ehrlich S., Scherag S., Herpertz-Dahlmann B., Zipfel S., Herzog W., de Zwaan M., Burghardt R. (2011). Genetic variation of the ghrelin activator gene ghrelin o-acyltransferase (GOAT) is associated with anorexia nervosa. J. Psychiatr. Res..

[117] Legrand R., Lucas N., Breton J., Azhar S., do Rego J.C., Déchelotte P., Coëffier M., Fetissov S.O. (2016). Ghrelin treatment prevents development of activity based anorexia in mice. Eur. Neuropsychopharmacol..

[118] Takagi K., Legrand R., Asakawa A., Amitani H., François M., Tennoune N., Coëffier M., Claeyssens S., do Rego J.C., Déchelotte P. (2013). Anti-ghrelin immunoglobulins modulate ghrelin stability and its orexigenic effect in obese mice and humans. Nat. Commun..

[119] Broglio F., Gianotti L., Destefanis S., Fassino S., Abbate Daga G., Mondelli V., Lanfranco F., Gottero C., Gauna C., Hofland L. (2004). The endocrine response to acute ghrelin administration is blunted in patients with anorexia nervosa, a ghrelin hypersecretory state. Clin. Endocrinol..

[120] Miljic D., Pekic S., Djurovic M., Doknic M., Milic N., Casanueva F.F., Ghatei M., Popovic V. (2006). Ghrelin has partial or no effect on appetite, growth hormone, prolactin, and cortisol release in patients with anorexia nervosa. J. Clin. Endocrinol. Metab..

[121] Weikel J.C., Wichniak A., Ising M., Brunner H., Friess E., Held K., Mathias S., Schmid D.A., Uhr M., Steiger A. (2003). Ghrelin promotes slow-wave sleep in humans. Am. J. Physiol. Endocrinol. Metab..

[122] Miljic D., Djurovic M., Pekic S., Doknic M., Stojanovic M., Milic N., Casanueva F.F., Ghatei M., Popovic V. (2007). Glucose metabolism during ghrelin infusion in patients with anorexia nervosa. J. Endocrinol. Investig..

[123] Hotta M., Ohwada R., Akamizu T., Shibasaki T., Takano K., Kangawa K. (2009). Ghrelin increases hunger and food intake in patients with restricting-type anorexia nervosa: A pilot study. Endocr. J..

[124] Arai T., Maejima Y., Muroya S., Yada T. (2013). Rikkunshito and isoliquiritigenin counteract 5-HT-induced 2C receptor-mediated activation of pro-opiomelanocortin neurons in the hypothalamic arcuate nucleus. Neuropeptides.

[125] Sadakane C., Muto S., Nakagawa K., Ohnishi S., Saegusa Y., Nahata M., Hattori T., Asaka M., Takeda H. (2011). 10-Gingerol, a component of rikkunshito, improves cisplatin-induced anorexia by inhibiting acylated ghrelin degradation. Biochem. Biophys. Res. Commun..

[126] Ohnishi S., Watari H., Kanno M., Ohba Y., Takeuchi S., Miyaji T., Oyamada S., Nomura E., Kato H., Sugiyama T. (2017). Additive effect of rikkunshito, an herbal medicine, on chemotherapy-induced nausea, vomiting, and anorexia in uterine cervical or corpus cancer patients treated with cisplatin and paclitaxel: Results of a randomized phase II study (JORTC KMP-02). J. Gynecol. Oncol..

[127] Fazeli P.K., Lawson E.A., Faje A.T., Eddy K.T., Lee H., Fiedorek F.T., Breggia A., Gaal I.M., DeSanti R., Klibanski A. (2018). Treatment with a ghrelin agonist in outpatient women with anorexia nervosa: A randomized clinical trial. J. Clin. Psychiatry.

